# Supplementary figures and images for: The microbiota of Drosophila suzukii influences the larval development of Drosophila melanogaster
Source: PeerJ. 2019 Nov 19;7:e8097. doi: 10.7717/peerj.8097 (PMC6873876; doi:10.7717/peerj.8097)

Figure S1

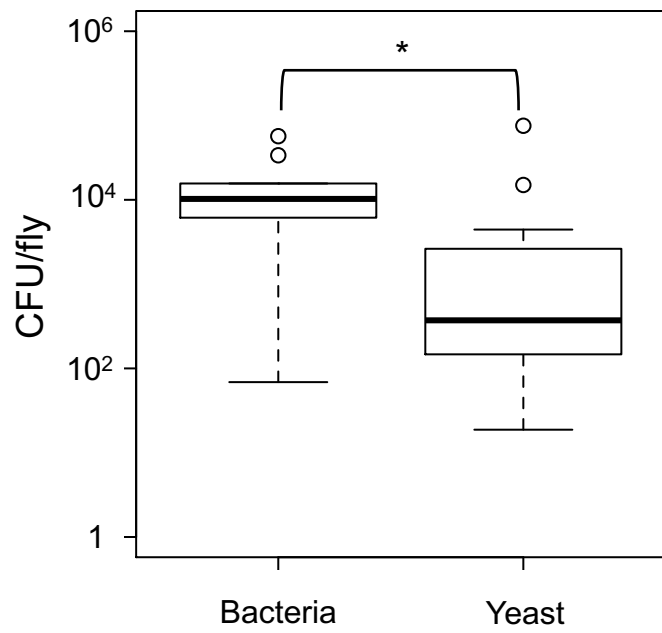

Supplement: Figure S1 — Field-caught flies were homogenized and plated on selective media to culture bacteria and yeasts separately. In each whisker box plot, the box delineates the first and third quartiles, the dark line is the median, and the whiskers show the range (minus outliers, which appear as circles). Bacterial colony forming units (CFU) per fly were significantly higher than that for yeasts (Mann–Whitney, P < 0.05, n = 15). [file peerj-07-8097-s002.pdf]

Figure S2

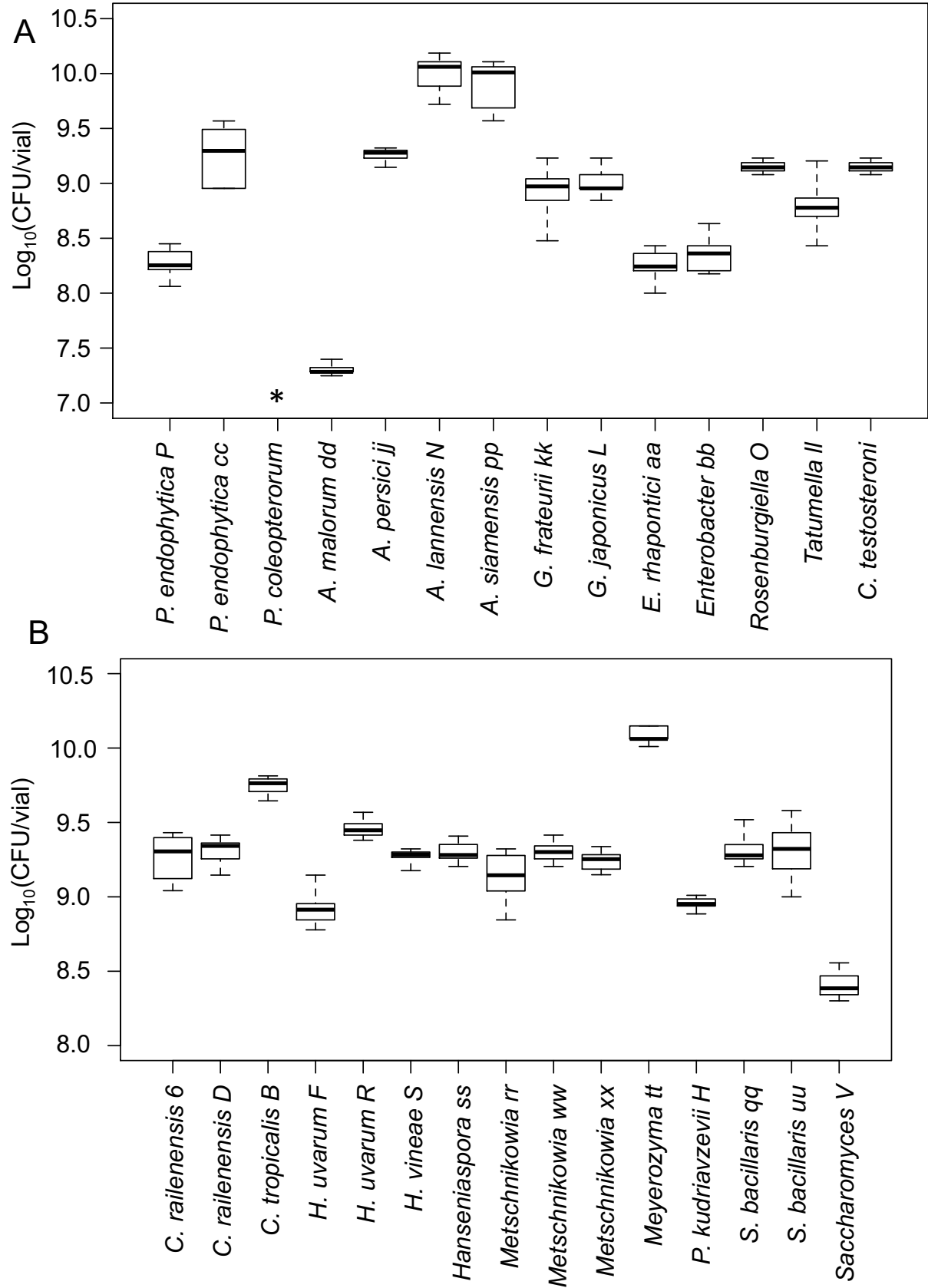

Supplement: Figure S2 — Bacteria (A) and yeasts (B) were quantified by serial dilution and plating on YPD. In each whisker box plot, the box delineates the first and third quartiles, the dark line is the median, and the whiskers show the range. *no CFU were recovered for P. coleoperorum. [file peerj-07-8097-s003.pdf]

Figure S3

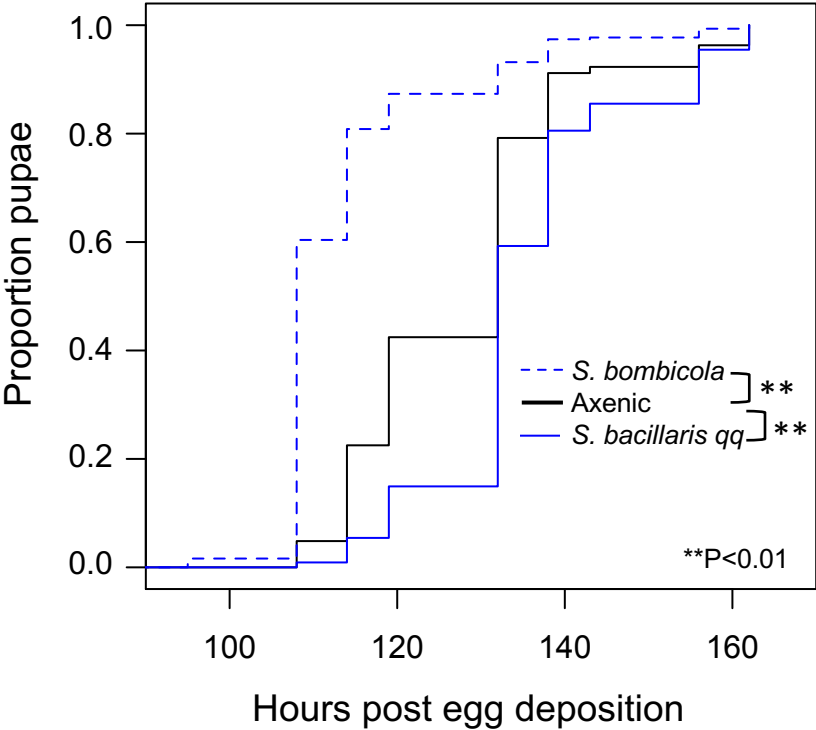

Supplement: Figure S3 — Kaplan–Meier plots depict the survival functions estimating the probability of pupariation at each time point after egg deposition based on observations of gnotobiotic Drosophila mono-associated with the yeast indicated in the inset legend. Each treatment was significantly different from the others in a Cox mixed-effects model, P < 0.01, n = 221 to 351 from two independent experiments. [file peerj-07-8097-s004.pdf]

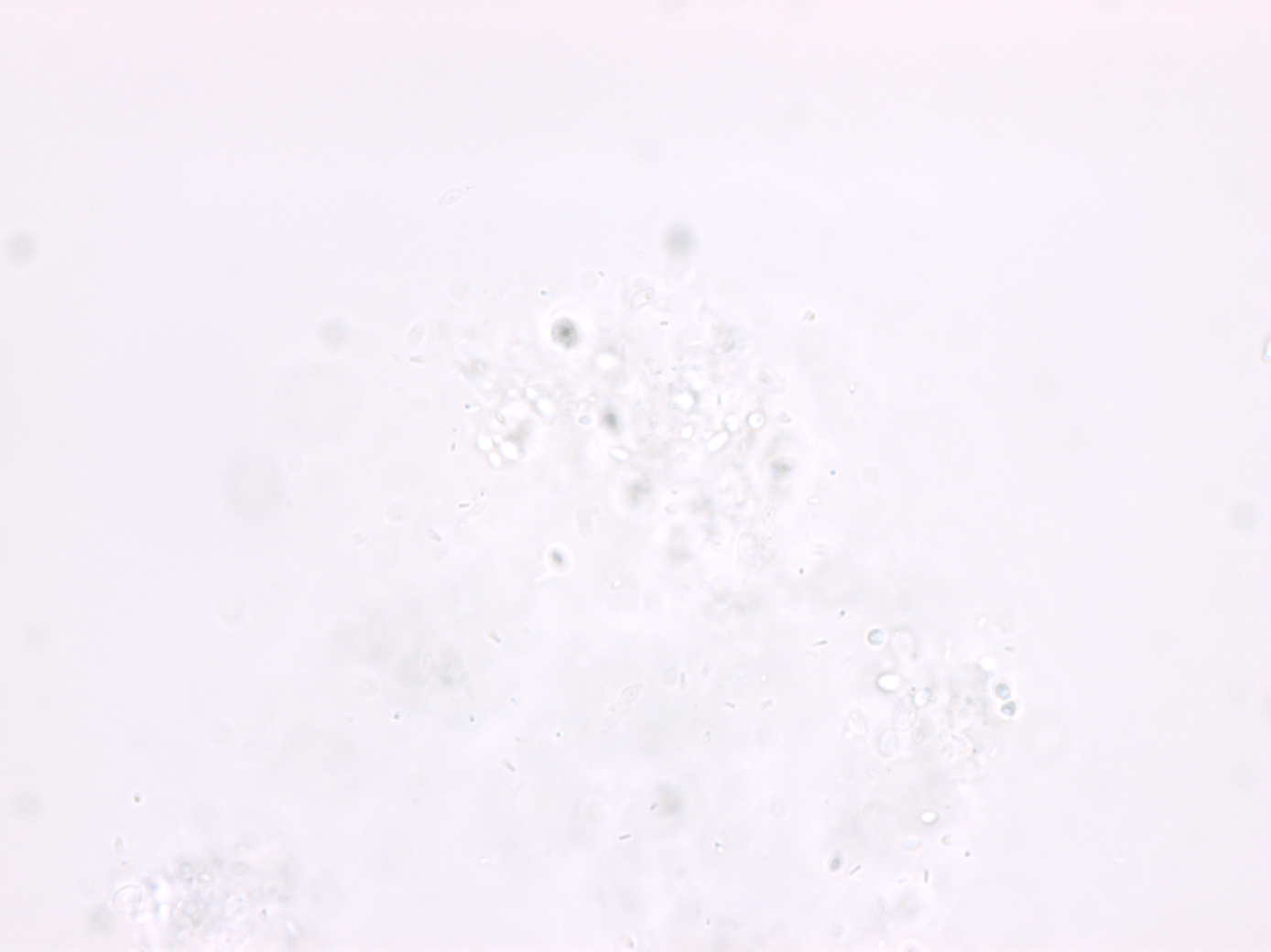

Supplement: Figure S6A [file peerj-07-8097-s005.tiff]

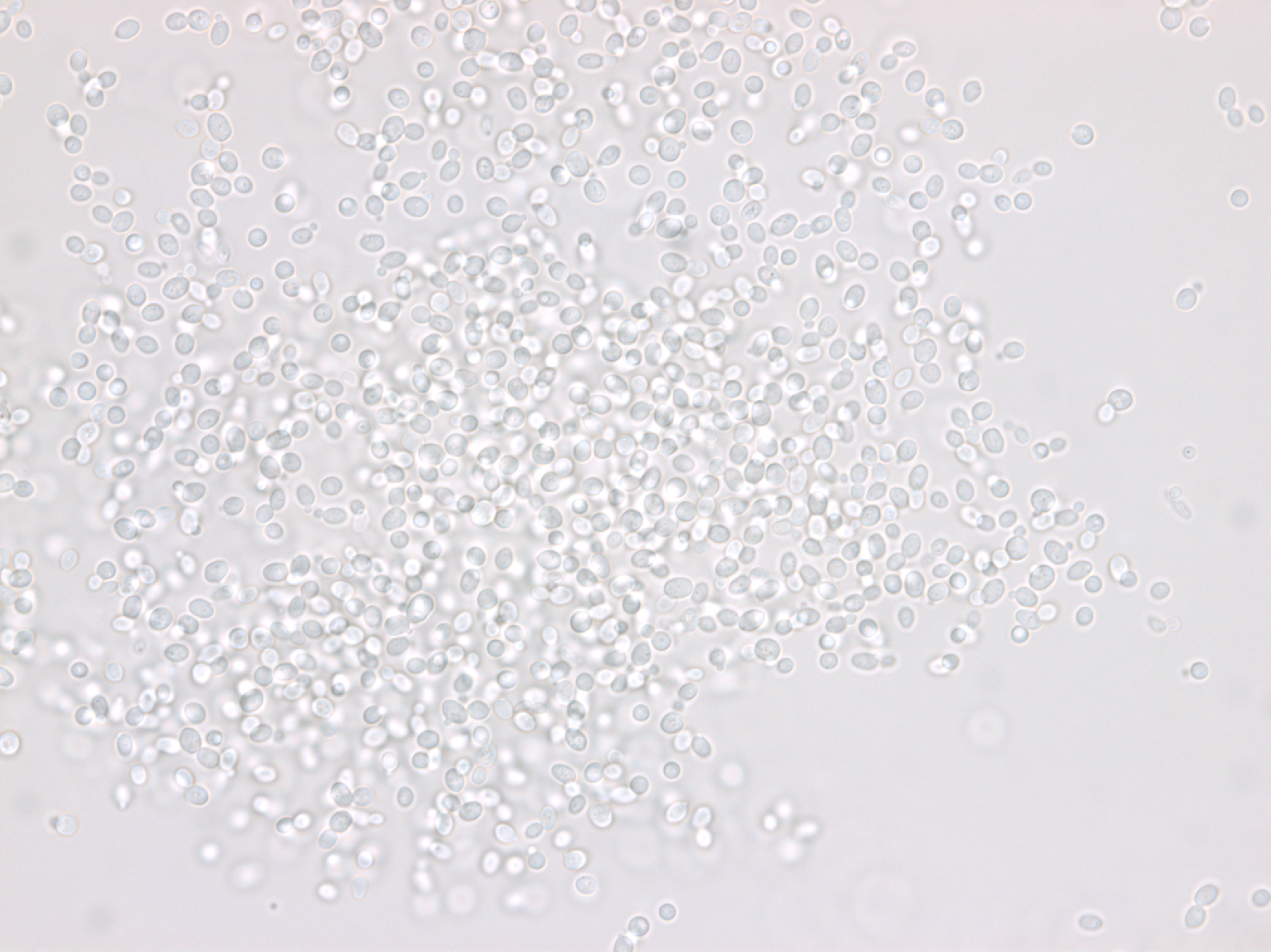

Supplement: Figure S6B [file peerj-07-8097-s006.tiff]

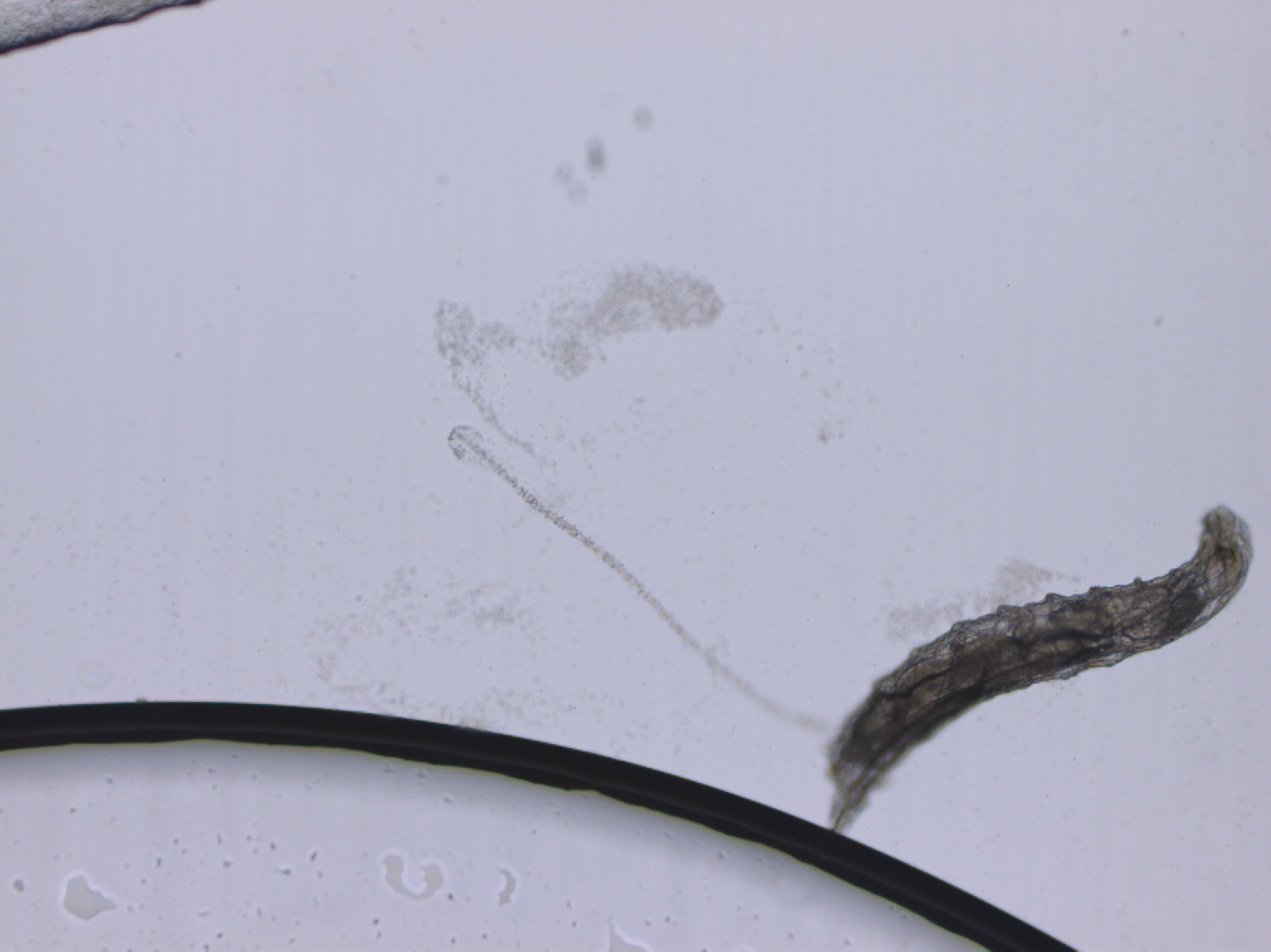

Supplement: Figure S6C [file peerj-07-8097-s007.tiff]

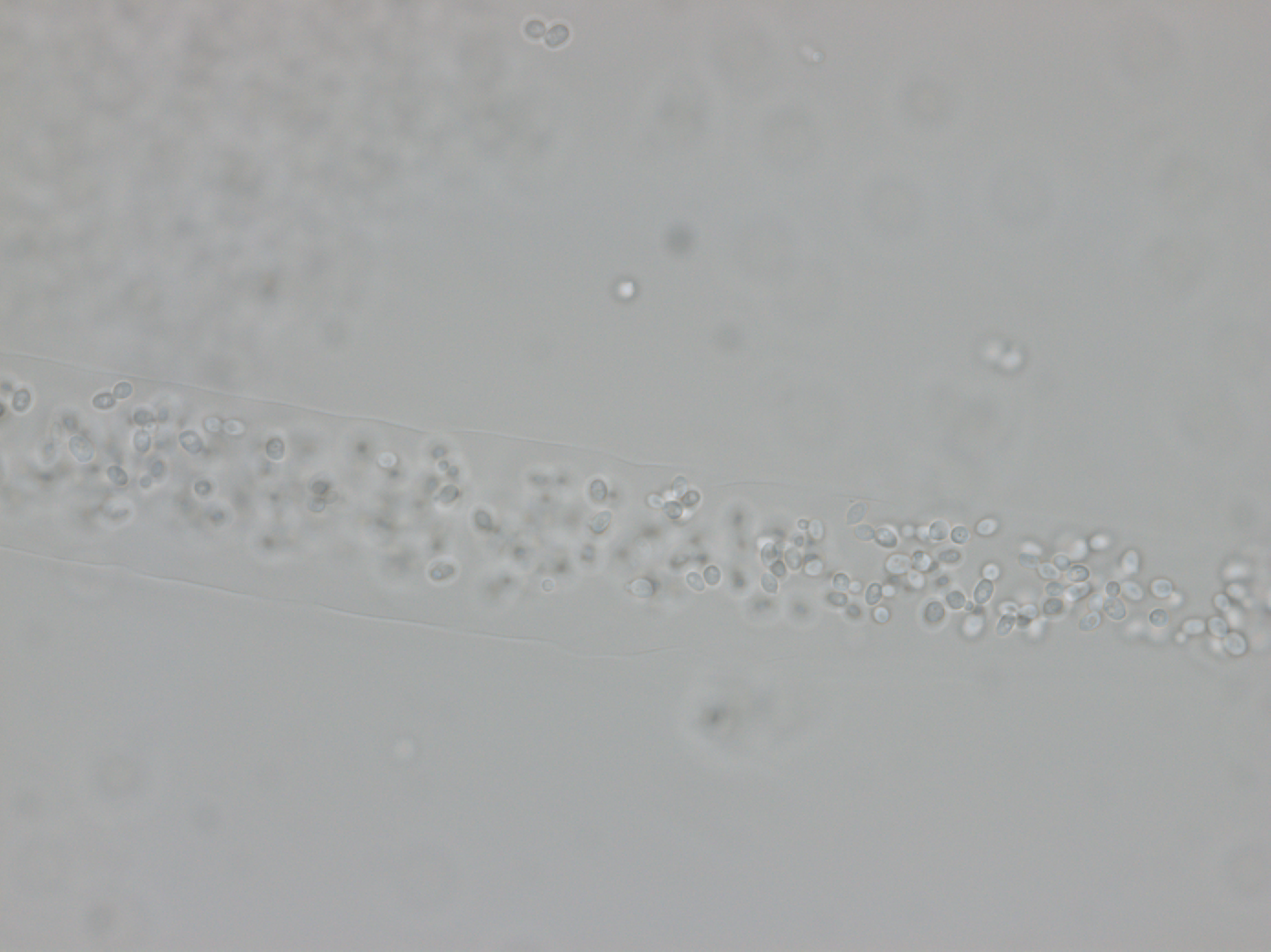

Supplement: Figure S6D [file peerj-07-8097-s008.tiff]
